# Correlation and Reliability Analyses among the Upper Cervical Rotation-Flexion Test, Upper Cervical Flexion-Extension Test, and Upper Cervical Flexion Angle Measurement Using Radiography

**DOI:** 10.3390/ijerph17145262

**Published:** 2020-07-21

**Authors:** KwangSun Do, JaeSung Choi, JaeEun Kim, JongEun Yim

**Affiliations:** 1 Department of Physical Therapy, Graduate School of Sahmyook University, Seoul 01795, Korea; kwangsun4988@gmail.com (K.D.); after214@naver.com (J.C.); 2 Catholic Kwandong University International St. Mary’s Hospital, Incheon 22711, Korea; 3 Department of Physical Therapy, Gumi University, Gumi-si 39213, Gyeongsangbuk-do, Korea; cosmicboi@nate.com

**Keywords:** upper cervical spine, atlanto-occipital joint, physical examination, range of motion

## Abstract

(1) Background: The cervical rotation-flexion test is one method of measuring the range of motion of the upper cervical spine; however, this test has not been investigated in detail. The aim of this study was to investigate the reliability and concurrent validity of the upper cervical rotation-flexion test. (2) Methods: Twenty-five healthy individuals (13 women and 12 men) participated in this study. The participants underwent radiography, the upper cervical flexion-extension test, and the upper cervical rotation-flexion test in a sitting position while wearing a cervical goniometer to measure the upper cervical flexion angle. Three experienced physical therapists administered the upper cervical rotation-flexion test using the cervical device, twice for each participant. Inter-rater and intra-rater reliabilities were evaluated using the intraclass correlation coefficient (95% confidence interval). (3) Results: The inter-rater and intra-rater reliability values of the total scores were excellent. The results of the upper cervical rotation-flexion test significantly correlated with those of the radiographic evaluation of the upper cervical flexion angle (*r* = 0.80, *p* < 0.001) and those of the upper cervical flexion-extension test (*r* = 0.77, *p* < 0.001). Significant correlations among the three test results were observed. (4) Conclusions: The findings of this study suggest that the upper cervical rotation-flexion test is meaningful for independently measuring the upper cervical flexion angle.

## 1. Introduction

Headache disorders are related to low quality of life and reduced work productivity [1,2,3]. Cervicogenic headaches result from musculoskeletal dysfunction in the cervical region, mainly occurring in the upper three cervical segments [4]. Upper cervical dysfunction is associated with pain and restriction in the range of cervical motion. [5,6,7]. Cervicogenic headaches and other forms of chronic headache, such as tension-type headaches and migraines, show some overlapping symptoms [8]. Therefore, appropriate physical examination is necessary to identify the physical signs of cervical dysfunction, which can aid the differential diagnosis.

Radiographic imaging is the most objective method for examining cervical mobility [9]. Radiography has the advantage of providing accurate and objective data; however, it also has disadvantages that make its clinical use difficult, such as examination cost and health risks associated with overexposure to radiation in cases of repeated imaging [10,11,12].

Range of motion is a fundamental evaluation procedure with ubiquitous applications [13,14]. Cervical range of motion can be assessed in cases of cervical dysfunction, headache, and neck pain, and this physical examination method has high sensitivity and specificity in identifying headache and neck pain problems [15]. However, because the cervical range of motion test examines the motion of the entire cervical spine, it is difficult to only determine the movement of the upper cervical joints with this method.

Two methods were reported in the literature for examining the upper cervical flexion motion. The first is the upper cervical flexion-extension (UCFE) test [16], the reliability of which has been measured in previous studies. Its inter-rater reliability was reported to be high, with an intraclass correlation coefficient (ICC) above 0.89. This test measures the flexion-extension angle of the upper cervical spine with the participant sitting on a chair and leaning against the wall to limit the movement of the lower cervical and upper thoracic vertebrae [16]. A recent study reported a moderate correlation (*r* = 0.62) between the results of the upper cervical flexion-extension test and the neck disability index in patients with cervical pain [17]. However, this method has limitations in measuring just the upper cervical spine. Throughout the testing, even though each volunteer is instructed to sit upright and keep their upper thoracic spine in contact with the wall, the motion of the lower cervical and thoracic spine cannot be eliminated. The second method is the upper cervical rotation-flexion (UCRF) test, which was introduced as a method for only examining the range of motion of the upper cervical spine in orthopedic physical therapy [18]. Cook et al. introduced the UCRF as a method for separately examining the range of motion of the upper cervical spine [19]. The UCRF test includes fully rotating the cervical spine and asking the patient to nod their head. If this test reveals limited range of motion, or functional dysfunction of C0–1 joint mobility may be suspected [19]. 

Although the literature reports that UCRF tests are widely used in clinical practice, their reliability and specificity in measuring only the upper cervical spine have not been adequately studied. Therefore, we analyzed the correlation between the UCRF test, upper cervical flexion angle measurement using radiography, and the UCFE test. We also investigated the intra-rater and inter-rater reliabilities of the UCRF test.

## 2. Materials and Methods

### 2.1. Participants

The participants were 25 healthy adults (12 men and 13 women) who were working at B hospital. They voluntarily participated in this experiment after receiving explanations of the purpose and process of the study.

A sample size of at least 15–20 individuals is considered acceptable for reliability studies collecting continuous data [20]. Individuals with pathological findings of the nervous or musculoskeletal systems, those who had been treated at a hospital for neck and shoulder issues within the last 6 months, and those with limited cervical movement owing to neck pain were excluded from the study. All subjects gave their informed consent for inclusion before they participated in the study. The study was conducted in accordance with the Declaration of Helsinki, and the protocol was approved by the Ethics Committee of the Experimental Process Research Ethics committee of the Sahmyook University (no. 2-7001793-AB-N-012018043HR).

### 2.2. Examiners

Three physiotherapists with at least 3 years of experience and familiarity with musculoskeletal testing performed the measurements. In addition, all examiners familiarized themselves with both the cervical range of motion instrument and the measurements of the upper cervical region before performing any evaluation. The examiners were blinded to each other’s results and independently recorded the data in separate spreadsheets.

### 2.3. Measurement Systems

We used a cervical goniometer (Cervical Range of Motion Instrument; MedNet Technologies, Elmont, NY, USA) that measured motion in three planes (flexion-extension, left-right lateral flexion, and left-right rotation) with three inclinometers. Capuano-Pucci et al. (1991) studied the reliability of cervical goniometers and reported high values of both inter-rater and intra-rater reliability.

### 2.4. Procedure

**Figure 1:**
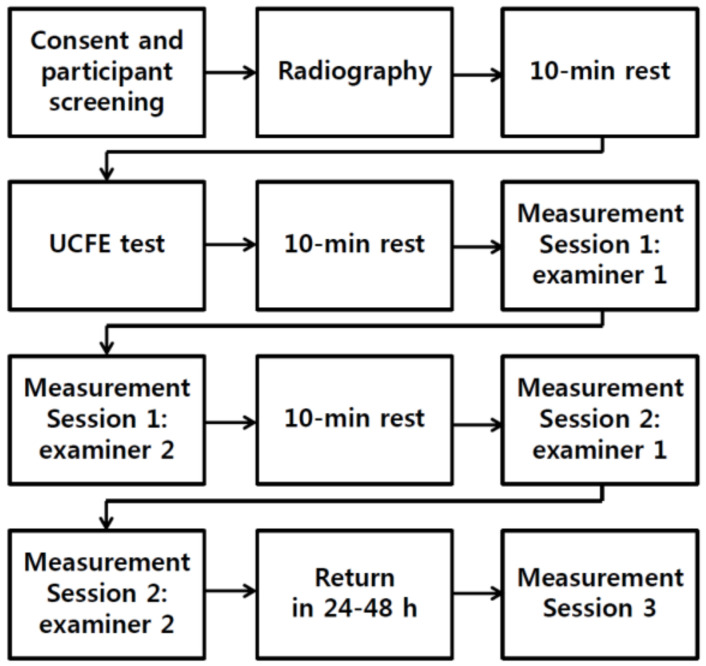
Flow chart of participant progression.

This study was designed as a cross-sectional study, and three tests were performed: (1) upper cervical flexion angle measurement using radiography, (2) the UCFE test, and (3) the UCRF test. On the first day, the participants were instructed about the study procedures before the experiment. Then, radiographs were taken. After a 10 min rest, the UCFE test was performed. Subsequently, the first session of UCRF tests was conducted by two examiners. After the measurements were taken by the two examiners, the subjects were allowed a 10 min rest before the next session. The second session was then performed in the same way as the first session. After 24–48 h, a third session was conducted by another examiner (Figure 1).

For all three tests, measurements were taken using a cervical goniometer with the participants sitting on a chair with a backrest. A standard board for postural analysis (Shisei PA200; The Big Sports Co., Dojimahama Kita-ku, Japan) with a longitudinal axis line was used to indicate the center of measurement. The chair was centered on the longitudinal axis line. In the UCRF test, the flexion angle was measured after fixing the back of the head with a flat board after cervical rotation.

### 2.5. Measurement of Upper Cervical Flexion Angle Using Radiography

**Figure 2:**
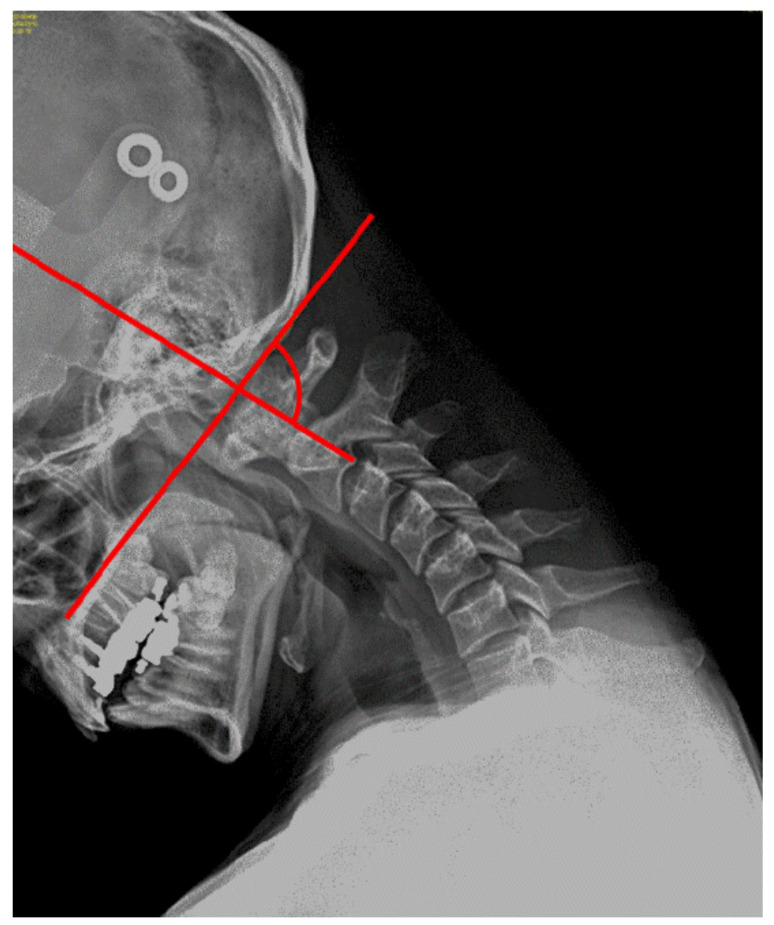
Measurement of upper cervical flexion angle using X-ray.

The upper cervical flexion angle was measured using an image processing program based on Java (ImageJ version 1.32; National Institutes of Health, Bethesda, MD, USA). The radiographs were taken in the sagittal plane, and the angle formed by the intersection of two tracings, including the McGregor plane (i.e., a line that connects the posterior nasal spine to the basiocciput) and the odontoid process plane (posterior surface of the odontoid process of C2), was measured (Figure 2) [21]. The neutral position of the upper cervical spine was measured by placing the external auditory meatus and acromion in a straight line, with the participant in a sitting position and leaning on the backrest while keeping the cervical goniometer at 0°. After taking radiographs in the neutral position, the cervical spine was flexed until a slight resistance was felt in the surrounding tissue without the motion of the thoracic spine. The upper cervical flexion angle was measured as the difference between the radiographic C0–1 angle in the neutral position and the radiographic C0–1 angle in the cervical flexion position. When the entire cervical spine is flexed, both the upper cervical spine and lower cervical spine are flexed, such that the angle of flexion of the upper cervical spine can be measured on radiographs.

### 2.6. UCFE Test

To prevent compensation during cervical flexion, the thoracic spine and both scapulae were placed as closely to the wall as possible. The participant was instructed to reach the manubrium of the sternum such that no tension was felt on the posterior neck, and the flexion angle was measured with a cervical goniometer [16].

### 2.7. UCRF Test

**Figure 3:**
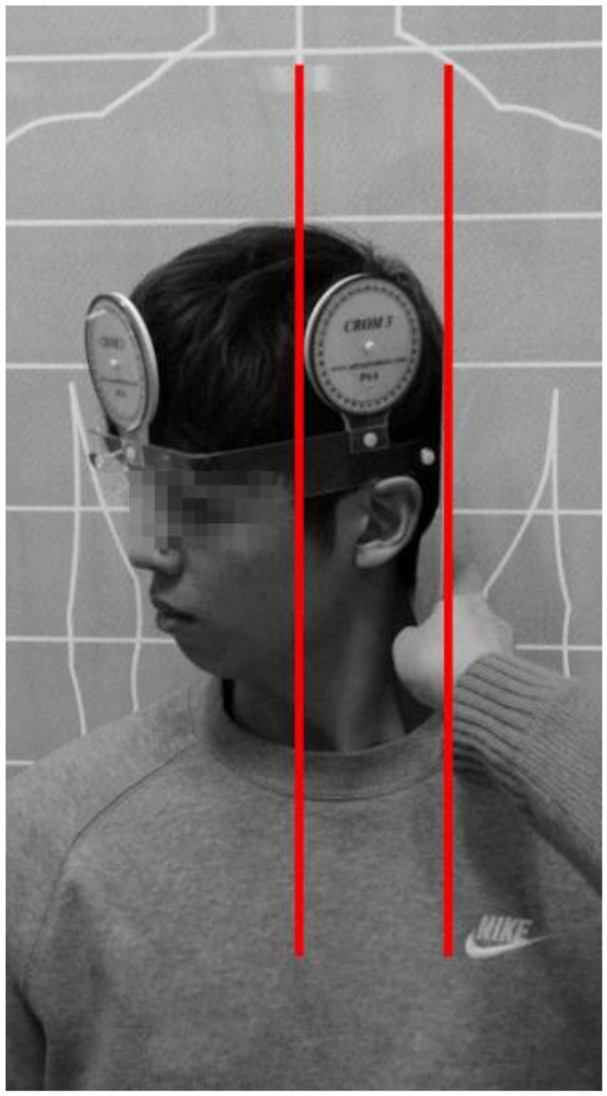
Upper cervical rotation-flexion test.

The UCRF test was performed to maximize the rotation of the cervical spine, and a plate was placed on the back of the participant’s head to prevent compensatory action. The plate was positioned parallel to the longitudinal axis line to establish the starting reference point. The participant was instructed to pull their chin to their clavicle until slight tension was felt in the cervical spine, and the flexion angle was measured using a cervical goniometer (Figure 3). The average value of left and right rotation-flexion angles was used.

### 2.8. Statistical Analysis

Statistical analyses were performed using SPSS version 18.0 (SPSS Inc., Chicago, IL, USA). The data were confirmed for normal distribution using the Shapiro–Wilk test. Pearson’s correlation coefficient was used to compare the correlation between the UCRF test, UCFE test, and the flexion angle measurement using radiography. Intra-rater and inter-rater reliabilities were determined using ICCs and associated 95% confidence intervals (CIs) [22], the use of which is a common approach to quantify the reliability of a measurement process [23]. Shrout and Fleiss (1979) suggested that ICC values > 0.75 indicate excellent reproducibility, values between 0.40 and 0.75 represent fair to good reliability, and values < 0.40 indicate poor reproducibility. Calculations were also made for the standard error of measurement (SEM; SEM = SD√1 − ICC $\mathrm{SD}\sqrt{1-\mathrm{ICC}}$) and the minimal detectable change (MDC; MDC = SEM × 1.96 × √2 $\sqrt{2}$) [24]. The SEM was calculated to determine the measurement error, and the MDC was calculated to determine the minimum threshold of measurement to ensure that differences between measurements were real and outside of the error range. The values were considered reliable when the SEM was <15% of the mean of the measured value [25] and when the MDC was <15% of the highest score in the measured values [26].

## 3. Results

In total, 25 participants were assessed for upper cervical range of motion (13 women, 12 men; mean age, 32.04 ± 6.94 years; mean height, 167.16 ± 6.92 cm; and mean weight, 63.37 ± 9.76 kg).

### 3.1. Validity

The mean value of the upper cervical flexion angle was 8.84° ± 2.30° in the UCRF test, 10.32° ± 3.64° in the UCFE test, and 7.60° ± 2.71° in the radiographic measurement. Pearson’s correlation coefficient between the UCRF test and radiography was *r* = 0.80 (*p* < 0.001), between the UCRF and UCFE tests was *r* = 0.77 (*p* < 0.001), and between the UCFE test and radiography was *r* = 0.81 (*p* < 0.001). Pearson’s correlation coefficient revealed a strong and significant association among all three tests (>0.75) (Table 1).

**Figure 4:**
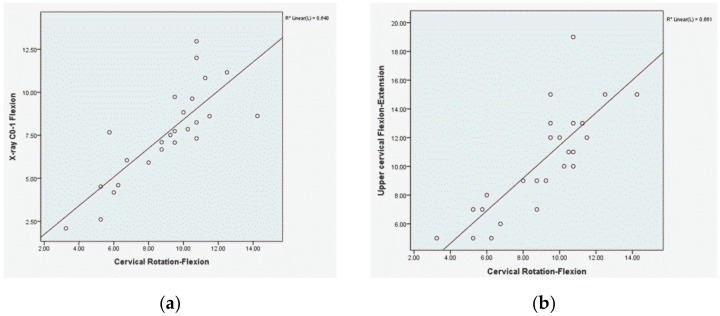
(**a**) Scatterplot showing the relationship between the upper cervical flexion angle using X-ray and the upper cervical rotation-flexion test (UCRF); (**b**) scatterplot showing the relationship between the upper cervical flexion-extension (UCFE) and UCRF tests.

The UCRF test and radiography showed a positive linear relationship (*r*^2^ = 0.64) (Figure 4). The UCRF and UCFE tests also showed a positive linear relationship (*r*^2^ = 0.66) (Figure 4).

### 3.2. Intra-Rater Reliability of the UCRF Test

The intra-rater reliability (ICC (95% CI)) values were 0.93 (0.85–0.97) for examiner 1, 0.94 (0.87–0.97) for examiner 2, and 0.95 (0.90–0.98) for examiner 3 (Table 2). The measurements of all three examiners showed excellent reproducibility. The SEMs and MDCs for examiners 1, 2, and 3 were 0.70, 0.54, and 0.58, and 1.95, 1.50, and 1.61, respectively. The measurement error was <15%; thus, the values were reliable.

### 3.3. Inter-Rater Reliability of the UCRF Test

The inter-rater reliability (ICC (95% CI)) values were 0.90 (0.82–0.95) for the first session and 0.95 (0.91–0.98) for the second session (Table 3). The ICC values for the first and second sessions for both examiners were in the excellent range (0.82–0.98).

The SEM values were 0.80° for first session and 0.48° for the second session. The MDC values were 2.24° for the first session and 1.35° for the second session. As the measurement error was <15%, the values were reliable.

## 4. Discussion

The aim of this study was to analyze the correlation between two goniometrically determined clinical measures of upper cervical flexion range of motion and a radiographic measurement of upper cervical flexion range of motion. The intra-rater and inter-rater reliabilities of the UCRF test were investigated.

A strong, significant correlation was found between the results of the UCRF test and the radiography results (*r* = 0.805, *p* < 0.001), demonstrating that the measurements taken in the UCRF test had criterion validity when compared with measurements from radiography with an objective and quantitative angle view. In addition, linear regression analysis revealed a value of *r*^2^ = 0.648, showing a positive linear relationship. The cervical spine is divided into the upper part (C0–2) and the lower part (C3–C7), which are mechanically different from each other [27]. Because the movement of the occipital bone and the first cervical vertebra is included in the entire cervical flexion movement, it is difficult to separate the general cervical range of motion in the evaluations [17]. Radiography is mainly used to examine the objective state of each vertebrae, and the range of motion of the upper cervical spine can be objectively quantified based on its anatomical location. However, due to the disadvantages such as increased expenses and overexposure to radiation in repeated radiographic examinations, re-evaluation for confirmation of the treatment effect during rehabilitation may be difficult [10,11,12]. Given these disadvantages, the UCRF test, which is clinically simple and practical, is used by many clinicians. However, despite its use in clinical practice, no studies have examined the reliability of this test and its correlation with other objective test methods. This study is meaningful as it is the first study, to the best of our knowledge, to compare the assessment of upper cervical flexion movement with objective radiographic examination.

We analyzed the correlation between the UCRF and UCFE tests. Pearson’s correlation coefficient analysis showed a strong correlation (*r* = 0.778, *p* < 0.001) between the UCRF and UCFE tests. In addition, linear regression analysis revealed a *r*^2^ of 0.661, indicating a positive linear relationship. The UCFE test is a functional test frequently used in clinical practice together with the UCRF test. It was found to have an intra-rater reliability of ICC > 0.89. In previous studies, there was a moderate correlation between the upper cervical flexion angle and the neck disability index when the UCFE test was performed in patients with headaches. However, there is no study on the criterion validity that serves as a reference for the upper cervical function test methods, and it has been suggested that radiological examination methods, such as radiography or magnetic resonance imaging, are necessary [17]. Accordingly, there was a need for correlation analysis studies between upper cervical function test methods and objective and quantitative radiological examinations.

Although the three methods were highly correlated, the mean angles were slightly different. The upper cervical flexion angle measured using radiography was the lowest (7.60° ± 2.71°), followed by that obtained by the UCRF test (8.84° ± 2.30°). The value in the UCFE test was the highest (10.32° ± 3.64°). The upper cervical flexion angle measured using radiography was the lowest most likely because this method measures the exact flexion angle of C0–1. The UCRF test limits the flexion of the cervical spine by increasing the tension of the atlanto-axial joint capsule, ligaments, and surrounding soft tissues as the C1–2 axis rotation increases. The lower cervical vertebrae rotate sequentially for each segment, limiting the flexion movement due to the increase in tension of tissues, such as ligaments and muscles located in the lower cervical spine. However, as the superior articular facet and occipital condyle of the C0–1 joint form deep concave-convex joints, the flexion-extension movement can be performed without being affected by axial rotation [28]. Anatomically, the maximal axial rotation of the cervical spine causes locking of the lower cervical spine, resulting in a separate movement of the upper cervical vertebrae when flexing after the maximal rotation of the cervical spine. Axial rotation restricts cervical flexion; however, it is thought that the average flexion angle is slightly higher than the upper cervical flexion angle on radiography because it cannot be completely restrained. The values from the UCFE test were the highest among the three tests. The UCFE test most likely caused more flexion movement because of compensatory action, as it was difficult to completely limit the motion of the lower cervical and upper thoracic spine.

All intra-rater ICC values of the UCRF test were >0.90. The SEM range for all examiners was 0.54°–0.70°, and the MDC was within the range of 1.50°–1.95°, which is a reliable level (<15%). This is a very good result indicating excellent intra-rater reliability. The inter-rater ICC values were 0.90 (0.82–0.95) for the first session and 0.95 (0.91–0.98) for the second session. Although the ICC values of the second session were higher and the 95% CI range was smaller and more reliable than those of the first session, both sessions were acceptable because they showed good reliability values of >0.90. The SEM and MDC were 0.80° and 2.24° in the first session and 0.48° and 1.35° in the second session, respectively, both of which were within the reliable level (<15%).

The limitations of this study were that the results cannot be generalized because the participants were healthy adult men and women without musculoskeletal problems. Previous studies were reported that flexion restriction of upper cervical are related to headaches. [29,30]. Therefore, future studies will need to investigate the upper cervical flexion angle in patients with cervical dysfunction, cervicogenic headaches, and migraines.

## 5. Conclusions

Our study showed that the UCRF test has strong reliability and validity. The results of this study suggest that the UCRF test may be used as an objective and quantitative method in evaluating upper cervical flexion mobility in the clinical setting.

## Figures and Tables

**Table 1:** Pearson’s correlation of upper cervical flexion angles between three tests.

	Mean ± SD	Pearson’s Correlation (*r* Value)		
		UCRF Test	UCFE Test	X-Ray
UCRF test	8.84 ± 2.30	1	0.77 *	0.80 *
UCFE test	10.32 ± 3.64	0.77 *	1	0.81 *
X-ray	7.60 ± 2.71	0.80 *	0.81 *	1

Abbreviations: UCRF, upper cervical rotation flexion; UCFE, upper cervical flexion extension. * *p* < 0.001.

**Table 2:** Intra-rater reliability for upper cervical rotation-flexion test.

	UCRF Test		SEM (°)	MDC (°)	ICC (95% CI)
	1st Session	2nd Session			
Rater A	9.10 ± 3.07	8.90 ± 2.25	0.70	1.95	0.93 **(0.85, 0.97)
Rater B	8.82 ± 2.39	8.86 ± 2.33	0.54	1.50	0.94 **(0.87, 0.97)
Rater C	8.86 ± 2.61	8.70 ± 2.64	0.58	1.61	0.95 **(0.90, 0.98)

Abbreviations: UCRF, upper cervical rotation flexion; ICC, intraclass correlation coefficient; SEM, standard error of measurement; MDC, minimal detectable change; ** *p* < 0.001.

**Table 3:** Inter-rater reliability for the upper cervical rotation-flexion test.

1st Session				2nd Session		
	ICC (95% CI)	SEM (°)	MDC (°)	ICC (95% CI)	SEM (°)	MDC (°)
UCRF test	0.90 ** (0.82, 0.95)	0.80	2.24	0.95 ** (0.91, 0.98)	0.48	1.35

Abbreviations: UCRF, upper cervical rotation flexion; ICC, intraclass correlation coefficient; SEM, standard error of measurement; MDC, minimal detectable change; ** *p* < 0.001.

## References

[1] Stovner L., Hagen K., Jensen R., Katsarava Z., Lipton R.B., Scher A., Steiner T., Zwart J.A. (2007). The global burden of headache: A documentation of headache prevalence and disability worldwide. Cephalalgia.

[2] Stovner L.J., Andree C. (2010). Prevalence of headache in Europe: A review for the Eurolight project. J. Headache Pain.

[3] Stovner L.J., Hagen K. (2006). Prevalence, burden, and cost of headache disorders. Curr. Opin. Neurol..

[4] Amiri M., Jull G., Bullock-Saxton J. (2003). Measuring range of active cervical rotation in a position of full head flexion using the 3D Fastrak measurement system: An intra-tester reliability study. Man. Ther..

[5] Biondi D.M. (2005). Cervicogenic headache: A review of diagnostic and treatment strategies. J. Am. Osteopath. Assoc..

[6] Sjaastad O., Fredriksen T., Pfaffenrath V. (1998). Cervicogenic headache: Diagnostic criteria. Headache J. Head Face Pain.

[7] Zito G., Jull G., Story I. (2006). Clinical tests of musculoskeletal dysfunction in the diagnosis of cervicogenic headache. Man. Ther..

[8] Sjaastad O., Bovim G. (1991). Cervicogenic headache. The differentiation from common migraine. An overview. Funct. Neurol..

[9] Edwards J.Z., Greene K.A., Davis R.S., Kovacik M.W., Noe D.A., Askew M.J. (2004). Measuring flexion in knee arthroplasty patients. J. Arthroplast..

[10] Anderson L.D., D’alonzo R.T. (1974). Fractures of the odontoid process of the axis. JBJS.

[11] Effendi B., Roy D., Cornish B., Dussault R., Laurin C. (1981). Fractures of the ring of the axis. A classification based on the analysis of 131 cases. Bone Jt. J..

[12] Levine A.M., Edwards C. (1985). The management of traumatic spondylolisthesis of the axis. J. Bone Jt. Surg. Am. Vol..

[13] Magee D.J. (2014). Orthopedic Physical Assessment-E-Book.

[14] Gajdosik R.L., Bohannon R.W. (1987). Clinical measurement of range of motion: Review of goniometry emphasizing reliability and validity. Phys. Ther..

[15] Jull G. (1997). Management of cervical headache. Man. Ther..

[16] Dhimitri K., Brodeur S., Croteau M., Richard S., Seymour C.J. (1998). Reliability of the cervical range of motion device in measuring upper cervical motion. J. Man. Manip. Ther..

[17] Ernst M.J., Crawford R.J., Schelldorfer S., Rausch-Osthoff A.-K., Barbero M., Kool J., Bauer C.M. (2015). Extension and flexion in the upper cervical spine in neck pain patients. Man. Ther..

[18] Donatelli R.A., Wooden M.J. (2009). Orthopaedic Physical Therapy-E-Book.

[19] Cook G., Burton L., Kiesel K., Bryant M., Torine J. (2010). Movement: Functional Movement Systems: Screening, Assessment, and Corrective Strategies.

[20] Lexell J.E., Downham D.Y. (2005). How to assess the reliability of measurements in rehabilitation. Am. J. Phys. Med. Rehabil..

[21] Chan C.A. (2007). A review of the clinical significance of the occlusal plane: Its variation and effect on head posture. Int. Coll. Craniomandib. Orthop. (Iccmo) Anthol..

[22] Trevethan R. (2017). Intraclass correlation coefficients: Clearing the air, extending some cautions, and making some requests. Health Serv. Outcomes Res. Methodol..

[23] Ionan A.C., Polley M.-Y.C., McShane L.M., Dobbin K.K. (2014). Comparison of confidence interval methods for an intra-class correlation coefficient (ICC). BMC Med Res. Methodol..

[24] Weir J.P. (2005). Quantifying test-retest reliability using the intraclass correlation coefficient and the SEM. J. Strength Cond. Res..

[25] Beckerman H., Roebroeck M., Lankhorst G., Becher J., Bezemer P.D., Verbeek A. (2001). Smallest real difference, a link between reproducibility and responsiveness. Qual. Life Res..

[26] Lu W.-S., Wang C.-H., Lin J.-H., Sheu C.-F., Hsieh C.-L. (2008). The minimal detectable change of the simplified stroke rehabilitation assessment of movement measure. J. Rehabil. Med..

[27] Bogduk N., Mercer S. (2000). Biomechanics of the cervical spine. I: Normal kinematics. Clin. Biomech..

[28] Neumann D.A. (2013). Kinesiology of the Musculoskeletal System-E-Book: Foundations for Rehabilitation.

[29] Hall T.M., Briffa K., Hopper D., Robinson K. (2010). Comparative analysis and diagnostic accuracy of the cervical flexion–rotation test. J. Headache Pain.

[30] Fernández-Mayoralas D.M., Fernández-de-las-Penas C., Palacios-Cena D., Cantarero-Villanueva I., Fernández-Lao C., Pareja J.A. (2010). Restricted neck mobility in children with chronic tension type headache: A blinded, controlled study. J. Headache Pain.

